# A Peptide-Fc Opsonin with Pan-Amyloid Reactivity

**DOI:** 10.3389/fimmu.2017.01082

**Published:** 2017-09-04

**Authors:** James S. Foster, Angela D. Williams, Sallie Macy, Tina Richey, Alan Stuckey, Daniel Craig Wooliver, Richa Koul-Tiwari, Emily B. Martin, Stephen J. Kennel, Jonathan S. Wall

**Affiliations:** ^1^Department of Medicine, University of Tennessee Medical Center, Knoxville, TN, United States; ^2^Department of Radiology, University of Tennessee Medical Center, Knoxville, TN, United States

**Keywords:** Fc-fusion, peptide p5, amyloidosis, AA amyloid, phagocytosis

## Abstract

There is a continuing need for therapeutic interventions for patients with the protein misfolding disorders that result in systemic amyloidosis. Recently, specific antibodies have been employed to treat AL amyloidosis by opsonizing tissue amyloid deposits thereby inducing cell-mediated dissolution and organ improvement. To develop a pan-amyloid therapeutic agent, we have produced an Fc-fusion product incorporating a peptide, p5, which binds many if not all forms of amyloid. This protein, designated Fcp5, expressed in mammalian cells, forms the desired bivalent dimer structure and retains pan-amyloid reactivity similar to the p5 peptide as measured by immunosorbent assays, immunohistochemistry, surface plasmon resonance, and pulldown assays using radioiodinated Fcp5. Additionally, Fcp5 was capable of opsonizing amyloid fibrils *in vitro* using a pH-sensitive fluorescence assay of phagocytosis. In mice,^125^ I-labeled Fcp5 exhibited an extended serum circulation time, relative to the p5 peptide. It specifically bound AA amyloid deposits in diseased mice, as evidenced by biodistribution and microautoradiographic methods, which coincided with an increase in active, Iba-1-positive macrophages in the liver at 48 h postinjection of Fcp5. In healthy mice, no specific tissue accumulation was observed. The data indicate that polybasic, pan-amyloid-targeting peptides, in the context of an Fc fusion, can yield amyloid reactive, opsonizing reagents that may serve as next-generation immunotherapeutics.

## Introduction

The systemic amyloidoses are a family of rare diseases wherein normally soluble proteins form insoluble amyloid fibrils that deposit in various organs throughout the body resulting in dysfunction and severe morbidity (1). More than 30 distinct proteins, with dissimilar secondary structure and function, have been identified as components of visceral amyloid fibrils. The most common form, immunoglobulin light chain-associated (AL) amyloidosis, is a heterogeneous disease with respect to both the primary structure of the light chains involved and the varied anatomic distribution of the deposits (2). Other major forms of systemic amyloidosis are associated with the deposition of transthyretin, either wild-type (WT) (wtATTR) or mutant forms (ATTR) (3), leukocyte chemotactic factor 2 (Alect2) (4), and the acute phase protein serum amyloid protein A (AA) (5). Amyloid deposits are structurally complex and diverse, being comprised principally of proteinaceous fibrils as well as cell-derived, hypersulfated heparan sulfate proteoglycans, such as perlecan, and serum proteins, notably serum amyloid P component (SAP). Deposition of amyloid results in architectural damage to organs and tissues, particularly heart, kidney, and nerves, which leads to progressive disruption of organ function (6). Removal of tissue amyloid deposits would lead to improved organ performance, enhanced quality of life and likely a concomitant increase in survival rates for patients with these disorders. Such a therapeutic paradigm has emerged with the development of amyloid-reactive monoclonal antibodies (mAbs) capable of specifically binding the deposits, thereby opsonizing the otherwise non-immunogenic amyloid and facilitating dissolution of the amyloid by cells of the immune system (7). Early reports from the clinical evaluation of three such antibodies (NEOD001, 11-1F4 and the anti-SAP mAbs) have demonstrated improvement in organ performance as evidenced by positive changes in function-related biomarkers (8–11). Thus, opsonization of tissue amyloid by passive immunotherapy has become an important new strategy for treating patients with systemic amyloidosis. However, given the complexity and diversity of systemic amyloid diseases, the current anti-fibril antibody therapies may likely only be effective for patients with AL and AA amyloidosis. This may not be true of the humanized SAP-reactive immunoglobulin, which, in principle, could be used treat many types of systemic amyloid regardless of the type of precursor protein from which the fibrils are formed, due to the ubiquitous presence of SAP in all amyloid deposits. However, the universal efficacy of treatment using SAP-reactive Ab has yet to be demonstrated; in a preliminary report, only ~30% of enrolled patients demonstrated an improved SAP scan, indicating dissolution of tissue amyloid (8). Therefore, we posit that, given the heterogeneity of these diseases, no single immunotherapeutic mAb will be effective for treating all patients, and production of alternative reagents that are capable of pan-amyloid opsonization remains an important goal.

We have developed a panel of synthetic, polybasic peptides that specifically bind to many forms of human amyloid, including AL, AA, ALect2, ATTR, Alzheimer’s disease-associated (Aβ), and islet amyloid polypeptide (AIAPP), found in patients with type 2 diabetes (12–14). One of these peptides, designated p5, is a non-natural 31-amino acid peptide with eight lysine residues spaced in a heptad repeat such that when an α-helical secondary structure is formed, the charged side chains align along one face of the helix (15). This conformation promotes electrostatic interactions with hypersulfated glycosaminoglycans, such as heparin, and with amyloid fibrils (16). We have shown that radioiodinated p5 rapidly binds amyloid deposits systemically in a murine model of AA amyloidosis with little evidence of binding to healthy organs and tissues (17). An elongated version of peptide p5, designated p5+14, is being translated for amyloid imaging (13); however, given the specific and pan-amyloid reactivity of these peptides, our goal now is to utilize them as therapeutic agents. To this end and to take advantage of the clinical efficacy of opsonizing mAb therapy in amyloid patients, we have developed a peptide p5-Fc fusion construct, designated Fcp5. Addition of the Fc to the peptide serves two notable functions: extension of the circulating half-life of p5 and provision of an immunologically active Fc moiety to mediate antibody-dependent cellular phagocytosis [ADCP; reviewed in Park et al. (18)]. Fusion of Fc domain proteins to biologically active proteins and receptors is an effective strategy to enhance the biological half-life by exploiting the pH-dependent interactions of the Fc with the neonatal Fc-receptor [FcRn (19, 20)]. An added advantage of the Fc-fusion proteins is that they generate multivalent reagents, thereby potentially enhancing avidity of the associated ligand for the target (21). The success of this approach is documented by the fact that numerous Fc-fusion proteins have been approved by the US Food and Drug Administration for indications such as rheumatoid arthritis (etanercept) and type 2 diabetes (dulaglutide) (22–24). Despite the increasing use of this strategy to enhance the half-life of small proteins and peptides, Fc fusion is not routinely employed as a means to generate immunologically active reagents capable of ADCP, which requires the use of specific Fc isotypes and glycoforms (25).

To generate a novel, immunotherapeutic reagent for targeting systemic amyloidosis, we synthesized a murine Fc-fusion construct that incorporated the synthetic pan-amyloid-reactive peptide p5 with a murine IgG2a Fc. The murine Fc was used in this preliminary fusion to allow proof of principle studies in mouse models of amyloidosis without the complication of immunogenicity of a human Fc component. In this study, we show that the Fcp5 protein can be readily produced and purified from cell culture medium, that the product retains the amyloid binding properties of the p5 molecule, and that the opsonizing function of the Fc is intact. When radiolabeled, Fcp5 exhibited specific binding to systemic amyloid *in vivo* with enhanced serum half-life relative to the p5 peptide.

## Materials and Methods

### Peptides and Antibodies

Peptide p5 was synthesized and purchased as a crude preparation (Anaspec, Fremont, CA, USA) and purified by reverse phase high performance liquid chromatography (RP-HPLC), lyophilized, and stored at −20°C. Before use, the peptide was hydrated in sterile PBS and the concentration determined by micro-BCA assay (Thermo-Pierce, Waltham, MA, USA). The Aβ(1–40), IAPP, and IAPP(Ile26Pro) peptides were purchased as 90% pure preparations (Anaspec), stored frozen, and prepared for use as previously described (26). The rVλ6Wil was produced as a recombinant protein in *E. coli* and purified from the periplasmic space extract by RP-HPLC, as described (27). MOPC 173 murine IgG2a was from Biolegend (San Diego, CA, USA) and murine IgG2a Fc fragment was purchased from Acros Biosystems (Newark, DE, USA). Murine 11-1F4 mAb was prepared and supplied in sterile PBS by SAIC (Frederick, MD, USA). Biotinylated goat anti-mouse IgG was purchased from Sigma-Aldrich (St. Louis, MO, USA) and the Iba-1-reactive rabbit pAb was from Wako (Richmond, VA, USA).

### Fibrils and Amyloid Extracts

Amyloid-like fibrils were prepared in sterile PBS from purified rVλ6Wil, Aβ(1–40) and IAPP, as previously described (26). The fibrils were isolated by centrifugation at 15,000 × *g* for 5 min, and the presence of fibrils was confirmed by measuring the fluorescence emission (490 nm; excitation = 450 nm) of thioflavin T added to ~5 μg of fibril preparation. Human amyloid extracts were prepared from autopsy-derived organs using a modified water floatation method, as described elsewhere (28). Murine liver homogenates were prepared as previously described (26).

### Cloning of Fcp5

The codon-optimized cDNA for peptide p5 (amino acid sequence: GGGYS KAQKA QAKQA KQAQK AQKAQ AKQAK Q), flanked by 5-amino acid (VTPTV) spacers, was purchased from Integrated DNA Technologies (Coralville, IA, USA). The cDNA was cloned into the Nco I and Bgl II sites of the pFUSE-mIgG2A-Fc vector (Invivogen, San Diego, CA, USA), *via* PCR-based In-Fusion cloning (Clontech, Mountain View, CA, USA), and in-frame with the IL-2 secretory leader and the CH2 and CH3 domains of the murine IgG2a heavy chain. The plasmid sequence was verified by sequencing, using standard techniques, at the University of Tennessee Molecular Biology Core Facility.

### Production and Purification of Fcp5 Protein

Fcp5 protein was produced by transient transfection of HEK293T/17 cells (ATCC, Manassas, VA, USA) in 100-mm tissue culture dishes using 6 µg of plasmid DNA with 20 µg of linear polyethylenimine (Polysciences, Warrington, PA, USA) per dish and culturing 9 days in DMEM/F12 (Lonza, Walkersville, MD, USA) with 2% (v/v) immunoglobulin-depleted fetal bovine serum (Thermo-Hyclone, Logan, UT, USA) and penicillin–streptomycin (Lonza), with media changes every 3 days. The collected cell supernatants were clarified by centrifugation at 1,500 × *g* for 10 min and the secreted Fcp5 purified by affinity chromatography using protein A-conjugated Sepharose (GE Healthcare, Pittsburg, PA, USA) with elution by 0.1 M glycine, pH 3.0, followed by dialysis in PBS and quantitation by Coomassie blue protein assay (Thermo-Pierce, Dallas, TX, USA). The integrity of the purified Fcp5 was documented by SDS-PAGE on native and reduced samples with staining by Coomassie brilliant blue, or by periodic acid Schiff staining for carbohydrate (Thermo-Pierce).

### *In Vitro* Binding Studies—Europium-Linked Immunosorbent Assay (EuLISA)

Bioactivity of the Fcp5, peptide p5, and Fc2a control was assessed using a EuLISA. The wells of a 96-well polystyrene microplate (Corning, Corning, NY, USA) were coated either with poly-l-lysine (Sigma-Aldrich) followed by low molecular weight heparin (Sigma-Aldrich), amyloid-like fibrils (0.83 µM), or monomeric forms of rVλ6Wil, Aβ(1–40) or human IAPP(Ile26Pro). Wells coated with fibrils or monomeric proteins were incubated overnight at 37 or 4°C, respectively. The wells were then treated with 200 µL of blocking buffer (PBS containing 1% bovine serum albumin; BSA) for 1 h at room temperature before washing with PBS and addition of the appropriate concentration of Fcp5, biotinylated-p5, or Fc2a in PBS with 1% (w/v) BSA and 0.05% (v/v) tween 20. Following a wash step, the bound Fcp5 and Fc2a were detected by addition of biotinylated goat anti-mouse IgG (Sigma-Aldrich). After washing with PBS/tween, wells were incubated with 100 µL of europium–streptavidin (Perkin Elmer, Waltham, MA, USA) and washed, followed by addition of 100 µL enhancement solution (Perkin Elmer) before measurement of time-resolved fluorescence emission using a Wallac Victor 3 plate reader (Perkin Elmer).

### Radioiodination

Briefly, Fcp5, p5, Fc2a, and mAb 11-1F4 were radioiodinated with 2 mCi iodine-125 (^125^I; Perkin Elmer) in the presence of 10 µg of chloramine T. The radiolabeled products were purified by size-exclusion gel filtration using either; Sephadex G-25 (PD10; GE Healthcare, Pittsburgh, PA, USA), Aca44 (Sigma-Aldrich), or Aca34 (Sigma-Aldrich) with a mobile phase of PBS containing 0.1% (w/v) gelatin, as previously described (29). The radiochemical yield was estimated by measuring the amount of ^125^I recovered in the purified pooled product relative to the amount of added ^125^I. The radiochemical purity and integrity of the purified product was assessed by SDS gel electrophoresis using 10% polyacrylamide gels followed by phosphor imaging (Cyclone Storage Phosphor System, Perkin Elmer, Shelton, CT, USA).

### *In Vitro* Binding Studies—Pulldown Assay

Binding of radioiodinated Fcp5, Fc2a, p5, and mAb 11-1F4 with amyloid-like fibrils, amyloid extracts, and murine liver homogenates was measured using a pulldown assay, as previously described (29).

### Surface Plasmon Resonance Measurements

Kinetic binding of Fcp5 and p5 to rVλ6Wil amyloid-like fibrils was assessed using surface plasmon resonance (BIAcore X; GE Healthcare, Pittsburgh, PA, USA). Experiments were performed as described previously (26) using a CM5 chip coated with rVλ6Wil fibrils (channel 1) and rVλ6Wil monomer (channel 2), as a control. Sensorgrams were recorded as the difference in resonance units of channel 1 minus channel 2. Seventy microliters samples of Fcp5 (2 µg/mL) or peptide p5 (0.2 µg/mL) were loaded and the data collected for 400 s (200 s binding-phase and 200 s dissociation-phase) with a flow rate of 20 µL/min. Binding and washout data were extracted from the sensorgram, aligned, and analyzed with the BIAevaluation software (Ver. 3) by fitting to the provided two-state binding algorithm with conformational change [*A* + *B* = *AB* = AB*].

### Immunohistochemistry and Histology

Formalin-fixed, paraffin-embedded tissues sections were cut at 6 µM and placed on Plus slides (Fisher Scientific, Norcross, GA, USA). Antigen retrieval was performed using Target Retrieval Solution™, pH 9 (Dako Corporation, Carpenteria, CA, USA), according to the manufacturer’s instructions. The tissue sections were then incubated with Fcp5 or control Fc2a protein at 0.15 µg/mL in PBS overnight at 4°C. The slides were then washed in water and further processed by antibody avidin–biotin detection of the bound Fc fragments (Elite Mouse IgG kit; Vector Laboratories, Burlingame, CA, USA) and development with diaminobenzidene reagent (ImmPACT™ Peroxidase Substrate kit; Vector laboratories). Tissue staining with Congo red (CR) and Iba-1-reactive pAb was performed as previously described (29, 30). Images were acquired using a Leica DMR microscope fitted with a SPOT-RT cooled CCD camera (Diagnostic Instruments, Sterling Heights, MI, USA). Image segmentation of the Iba-1-stained tissues was performed using the “count” feature in Image Pro Plus (v. 9.0.4) (Media Cybernetics, Rockville, MD, USA).

### *Ex Vivo* Phagocytosis Assay

The rVλ6Wil monomer and human ATTR extracts were labeled with the pH-sensitive dye, pHrodo green-STP ester (Life Technologies), according to manufacturer’s instructions. Amyloid-like fibrils were prepared by angled shaking (225 rpm) of a solution of 100 µg pHrodo green-labeled rVλ6Wil monomer and 900 µg of unlabeled monomer for ~72 h at 37°C. Free pHrodo green dye was removed from the fibril and extract preparations by centrifugation at 10,000 × *g* for 15 min and washing in PBS. For the phagocytosis assay, ~1 × 10^6^ RAW 264.7 cells (ATCC) were added to each well of a 12-well culture dish, containing a glass coverslip, and incubated overnight. The following day, 4 µg of pHrodo green-labeled human ATTR extract or rVλ6Wil fibril preparation was mixed with 20 µg of Fcp5, MOPC2a, Fc2a, or, as a negative control, an equal volume of PBS. The sample was incubated for 30 min, unbound protein removed by washing each preparation in PBS with 2× centrifugation at 10,000 × *g* and the treated fibrils or extract added to the wells containing RAW 264.7 cells.

Following a 110-min incubation at 37°C, Hoescht 33342 dye (Life Technologies) was added to each well to stain the cell nuclei, and the mixture was incubated for a further 10 min. The coverslips were then washed with cell culture medium and live-mounted on a slide for image acquisition using a Leica DMR 500 epifluorescent microscope fitted with a SPOT-RT digital camera and blue or green fluorescence filter sets. Image segmentation was performed using Image Pro Plus software and the area of green fluorescence measured and expressed per cell (phagocytosis index) using 4 or 6 low magnification (5× objective) fields of view (FOV) from each coverslip. Approximately 2,000 cells were analyzed for each sample.

### Biodistribution Measurements

*In vivo* biodistribution and microautoradiography were performed, as previously described (13), in H2-Ld-huIL-6 Tg Balb/c mice with systemic AA amyloid deposition at 5 wk post IV injection of 100 µg amyloid-enhancing factor [AEF (31)]. Amyloid-free WT mice served as the control. Mice were administered, IV in the lateral tail vein, ~100 μCi ^125^I-Fcp5 [either 3 µg bolus (pharmacokinetics) or 100 µg bolus (biodistribution) of Fcp5 with 10% (w/w) ^125^I-Fcp5] in a 200 µL-volume of sterile PBS containing 0.1% gelatin. The Fcp5-injected mice were left from 1 h and up to 144 h postinjection before being euthanized by isoflurane inhalation overdose. Thereafter, the organs were harvested at necropsy for biodistribution and microautoradiographic analyses, as described (13).

Measurement of serum radioactivity was performed using cohorts of three mice bled at 1, 4, and 24 h post IV injection of ~30 μCi ^125^I-Fcp5. Blood was obtained from the retro-orbital sinus immediately before euthanasia. The blood was allowed to clot at 37°C for 15 min before being centrifuged at 15,000 × *g* for 2 min and serum collected.

### Ethics Statement

All patient-derived tissue samples were used in accordance with an Institutional Review Board-approved application. Animal studies were carried out in strict accordance with a protocols approved by the University of Tennessee Institutional Animal Care and Use Committee. All procedures were approved by the IACUC and were performed in accordance with the guidelines provided by OLAW and the Guide for the Care and Use of Laboratory Animals. The University of Tennessee Medical Center animal program is AAALAC-i-accredited.

### Statistical Methods

The binding of Fcp5 to substrates are shown as the mean ± SD of six replicates. Data points for the phagocytosis assay were compared between each respective using ANOVA with Dunnett correction for multiple comparisons; mean ± SD, *n* = 3. Evaluation of the macrophage distributions were performed using a two-tailed *t*-test with a significance value of *p* < 0.05. Analyses were performed using Prism ver. 6.07 (Graphpad Inc., La Jolla, CA, USA).

## Results

### Fcp5 Production and Characterization

**Figure 1:**
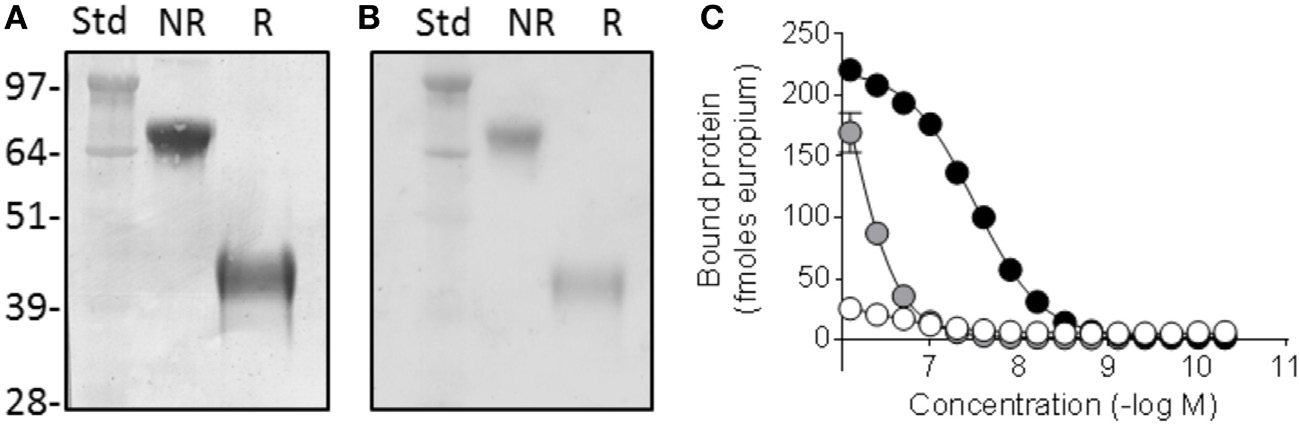
Fcp5 is secreted as a glycosylated dimer with heparin-binding activity. **(A)** Coomassie-stained SDS-PAGE gel showing dimeric construct in the non-reduced (NR) form which is reduced to monomer when reduced (R). **(B)** Glycosylation of Fcp5 shown by periodic acid Schiff-stained gel. **(C)** Heparin binding was observed with Fcp5 (black) and peptide p5 (gray) but not Fc2a control (white).

The Fcp5 expression construct was prepared by cloning the cDNA encoding peptide p5 with the addition of a five amino acid linker into, and in frame with, a vector encoding the murine IgG2aFc. Transient transfection of the Fcp5 encoding vector into HEK293T cells resulted in demonstrable Fcp5 protein production and secretion into the culture media at 48 h post transfection. The Fcp5 protein was subsequently purified from immunoglobulin-depleted cell culture medium supernatants of transiently transfected HEK293T cells by Protein A affinity chromatography with a yield of ~50 μg per 1 × 10^6^ cells. Analysis of the isolated Fcp5 by SDS-PAGE demonstrated a single band in the non-reduced form (Figure 1A) at ~85 kDa and, when reduced, at ~42 kDa consistent with the presence of a disulfide-linked glycosylated Fcp5 dimer (theoretical protein dimer Mw = 63,106) and monomer (Mw = 31,553), respectively. Staining the gel using periodic acid Schiff’s reagent indicated that Fcp5 was glycosylated, which likely accounted for the increase in the apparent molecular weight seen on the gel (Figure 1B).

Since p5 was developed as a heparin-reactive peptide, the bioactivity of purified Fcp5 was initially examined by studying the interaction with low molecular weight heparin (Figure 1C). The Fcp5 (black) bound heparin with an EC_50_ of ~30 nM, which was ~30-fold higher than the binding of the p5 peptide alone (~850 nM; gray). No reactivity of an IgG2a Fc fragment (Fc2a) control protein with heparin was observed (white).

### Fcp5 Reactivity with Amyloid-Like Fibrils and Monomeric Protein

**Figure 2:**
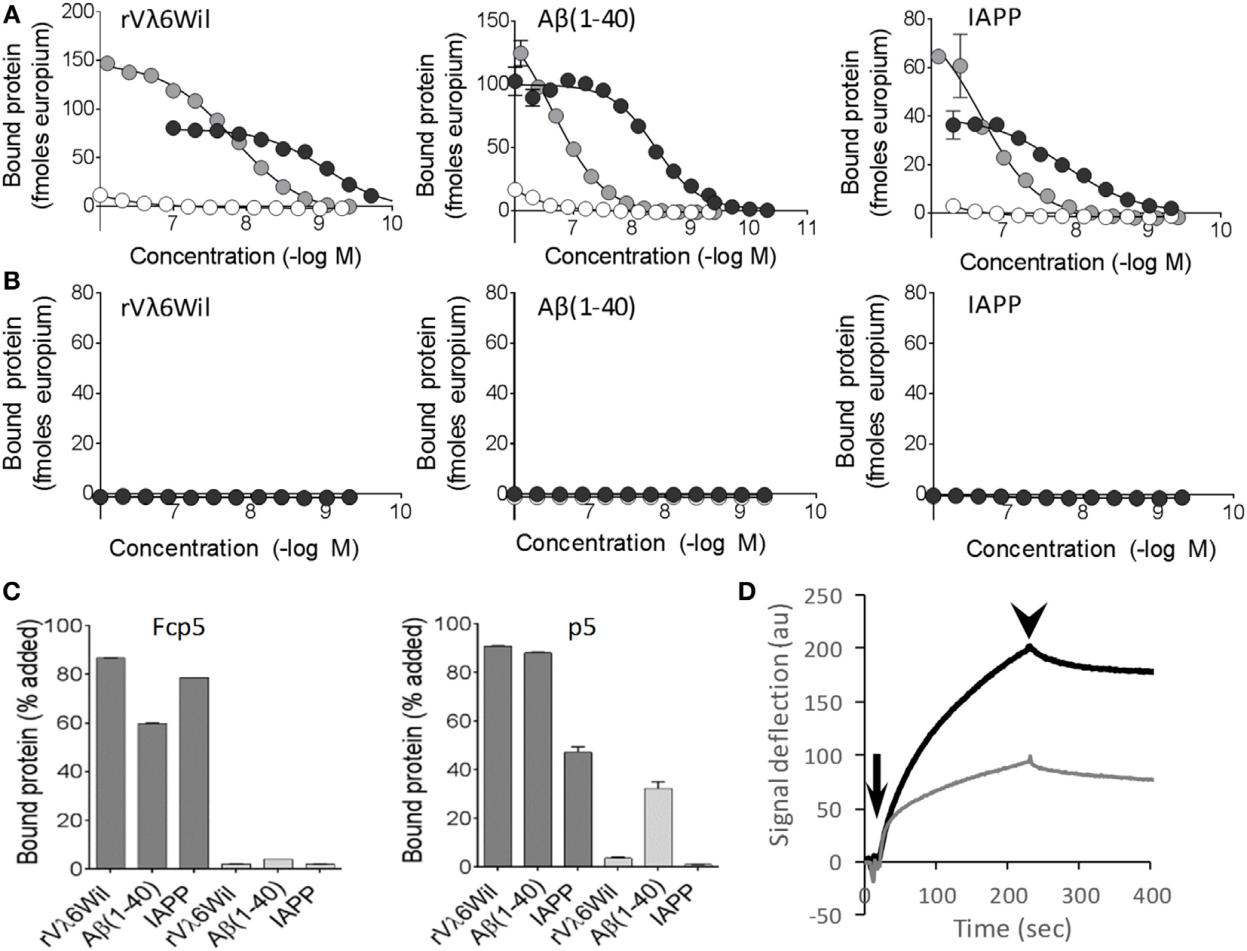
Fcp5 binds the three major forms of amyloid-like fibrils. **(A)** The Fcp5 construct (black) and peptide p5 (gray), but not Fc2a (white), bound surface-adsorbed amyloid-like fibrils composed of rVλ6Wil, Aβ(1–40), and IAPP. In each case, Fcp5 bound with greater affinity. **(B)** No reactivity of Fcp5 (black) or p5 (gray) was observed when non-fibrillar rVλ6Wil, Aβ(1–40), or human IAPP(Ile26Pro) were adsorbed to the surface of microplate wells. **(C)** Pulldown assays were performed using ^125^I-Fcp5 and peptide ^125^I-p5 in PBS (dark gray) and 1 M NaCl (light gray) to assess the importance of electrostatic interactions. **(D)** Kinetic binding of Fcp5 (black) and p5 (gray) to rVλ6Wil fibrils by surface plasmon resonance. Injection (arrow) and washout (arrowhead) phases occurred over 200 s.

The binding of Fcp5 with amyloid-like fibrils was compared to that of p5 using fibrils made from: an immunoglobulin light chain variable domain (rVλ6Wil), the Aβ(1–40) peptide, and IAPP (Figure 2). The Fcp5 (black), peptide p5 (gray), or Fc2a control (white) was added to fibrils immobilized in the wells of a microplate (Figure 2A). The Fcp5 bound rVλ6Wil, Aβ(1–40) and IAPP amyloid-like fibrils with estimated EC_50_ values of 1, 4, and 15 nM, respectively, which were higher affinities than those calculated for peptide p5 (14, 230, and 200 nM, respectively; Figure 2A). The control Fc2a, again, exhibited no detectable binding. Since only Fcp5 and p5 exhibited binding to the fibrils, the reactivity of these reagents was tested using monomeric forms of rVλ6Wil, Aβ(1–40) and a non-fibrillogenic form of human IAPP with a Pro substitution at position 26 (Figure 2B). In all cases, there was no reactivity of Fcp5 (black) or p5 (gray) with the non-fibrillar substrates.

The reactivity of Fcp5 and p5 with amyloid-like fibrils was further interrogated following radioiodination with iodine-125 (^125^I) in a “solution phase” pulldown assay, in PBS or in 1 M NaCl (Figure 2C). High salt milieu was used to examine the relative importance of electrostatic interactions in the binding. The interaction of ^125^I-labeled Fcp5 and p5 with the fibrils in PBS was comparable (Figure 2C, dark gray) and, with the exception of ^125^I-p5 binding to Aβ(1–40), the binding was completely abrogated in 1 M NaCl (Figure 2C, light gray).

Kinetic analysis of the interaction of Fcp5 (black) and p5 (gray) with rVλ6Wil fibrils was performed by using surface plasmon resonance (Figure 2D). Evaluation of the Fcp5 kinetic profile, using a 2-state binding algorithm, yielded a KD of ~3 nM and a signal deflection of ~200 au. Similar analysis of peptide p5 gave an approximately equivalent KD (~5 nM) but a deflection of only 100 au, consistent with the lower molecular weight of the p5 vs Fcp5.

### Human Tissue Amyloid Reactivity

**Figure 3:**
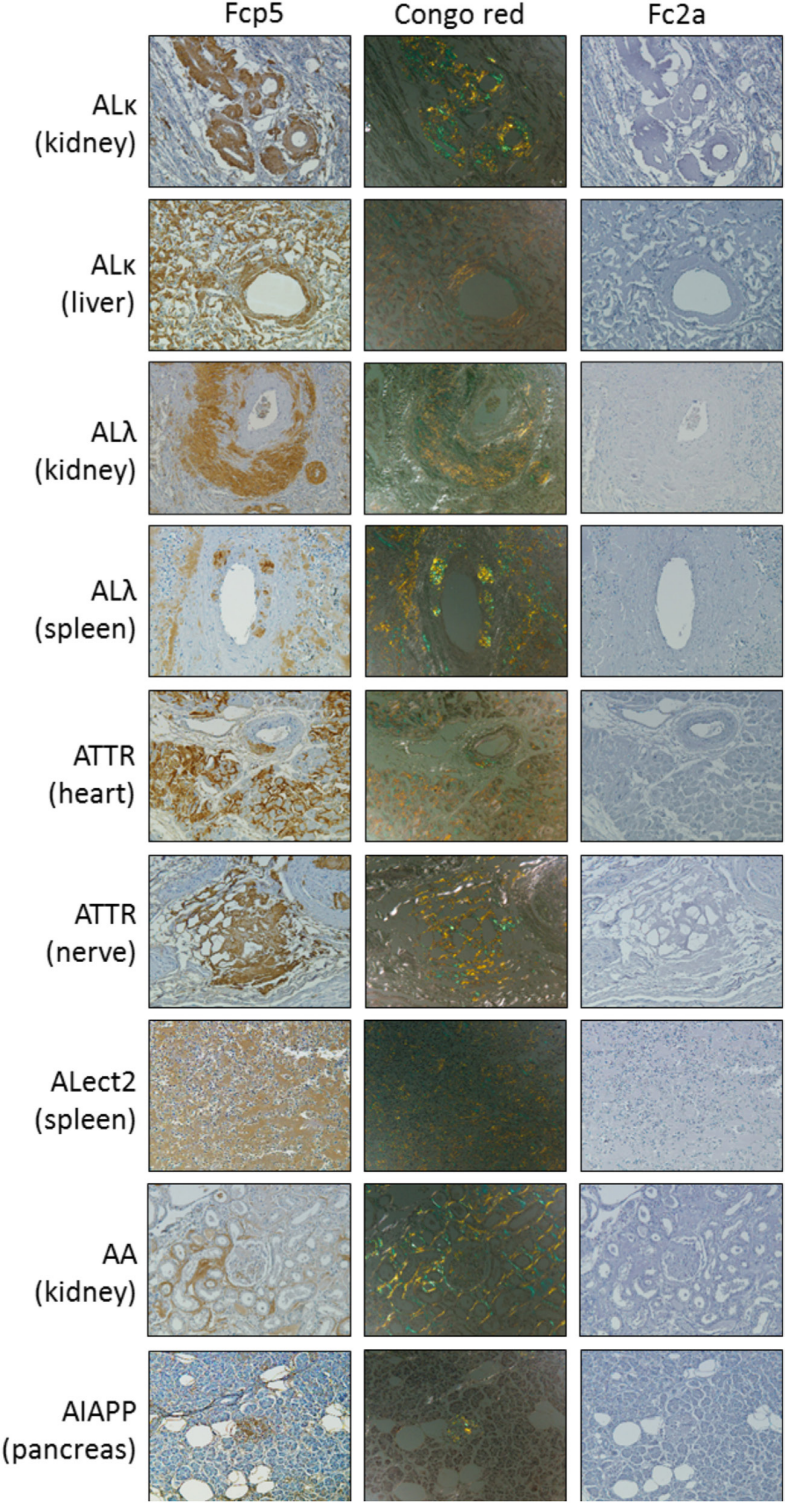
The Fcp5 specifically binds human amyloid deposits in formalin-fixed tissues. Fcp5 immunostained human ALκ, ALλ, ATTR, ALect2, AA, and AIAPP amyloid in formalin-fixed paraffin-embedded tissues as evidenced by the brown DAB staining which co-localized with the amyloid seen as green-gold birefringent material in Congo red-stained consecutive tissue sections. There was no binding of Fc2a. Original objective magnification 20×.

Peptide p5 can be used to immunostain many forms of human amyloid in tissue samples. To investigate whether this property was retained by the peptide in the context of the Fc fusion, formalin-fixed paraffin-embedded tissues obtained at autopsy from patients with AL (κ or λ), ATTR, ALect2, AA, or AIAPP-associated amyloidoses were immunostained using Fcp5 or with Fc2a as a negative control (Figure 3). In all cases, Fcp5 co-localized with amyloid deposits in the tissue, as evidenced by the brown diaminobenzidene staining that correlated with the tissue distribution of amyloid, shown as green birefringent material in CR-stained consecutive tissue sections.

### Amyloid Extract Reactivity

**Figure 4:**
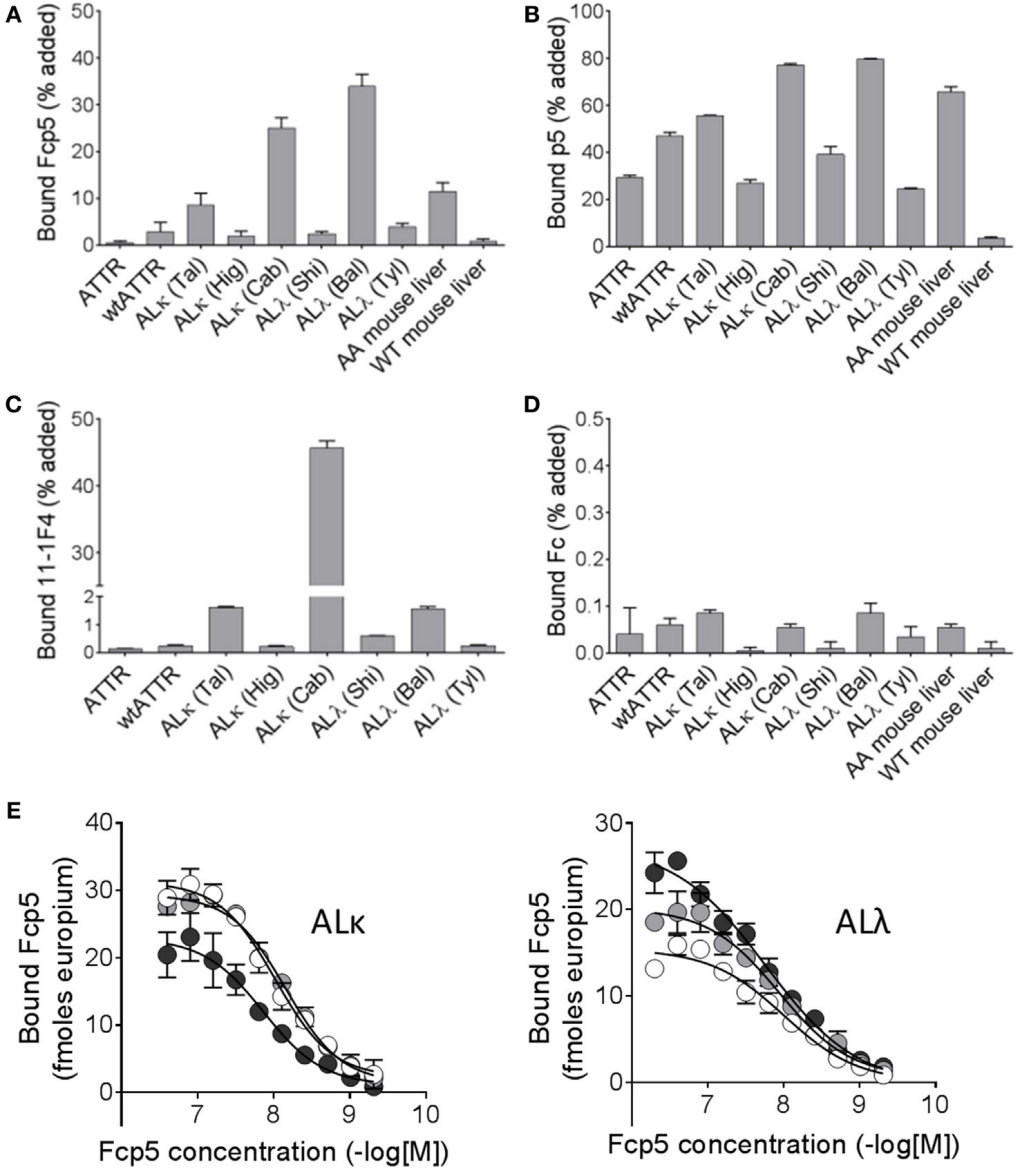
Radioiodinated Fcp5 binds human and murine amyloid extracts. Proteins Fcp5 **(A)**, p5 **(B)**, 11-1F4 **(C)**, and Fc2a **(D)** were radiolabeled and used in a pulldown assay to assess binding to human ATTR extracts, AL extracts, and mouse AA homogenates. **(E)** Binding of Fcp5 to surface-adsorbed human ALκ and ALλ amyloid extracts (three different patients per isotype) was assessed by europium-linked immunosorbent assay.

To further study the reactivity of Fcp5 with a more complex form of amyloid, pulldown experiments were conducted using radioiodinated Fcp5, p5, Fc2a, and the clinically relevant mAb, 11-1F4 (Figure 4), using as a substrate human ALκ, ALλ, and ATTR amyloid extracts as well as murine AA amyloid-laden liver homogenate, and a control healthy liver homogenate. As compared to the ^125^I-p5 peptide, the reactivity of ^125^I-Fcp5 with all amyloid extracts, with the exception of one ALκ (Cab) and one ALλ (Bal), was reduced by ~10-fold to 20-fold, but it remained positive (Figures 4A,B). The pattern of reactivity with each amyloid extract was similar for both ^125^I-Fcp5 and ^125^I-p5. The ^125^I-11-1F4 murine mAb did not avidly bind any of the amyloid extracts or tissue homogenates in this assay, with <2% of the ^125^I mAb bound in all cases, except ALκ (Cab; Figure 4C). The ^125^I-Fc2a, which served as a negative control, did not bind any of the substrates (<0.1% bound; Figure 4D).

A EuLISA was also used to evaluate the relative affinity of the binding of Fcp5 with human AL amyloid extracts (Figure 4E). Fcp5 was titrated into wells containing surfaced-adsorbed AL amyloid extracts (*n* = 3 ALκ and 3 ALλ). Binding to both ALκ and ALλ was similar in intensity with EC_50_ values, estimated from fitting a sigmoidal dose-response algorithm, ranged from 7 to 17 nM (mean EC_50_ = 11.5 nM, *n* = 6).

### *Ex Vivo* Opsonization Assay

**Figure 5:**
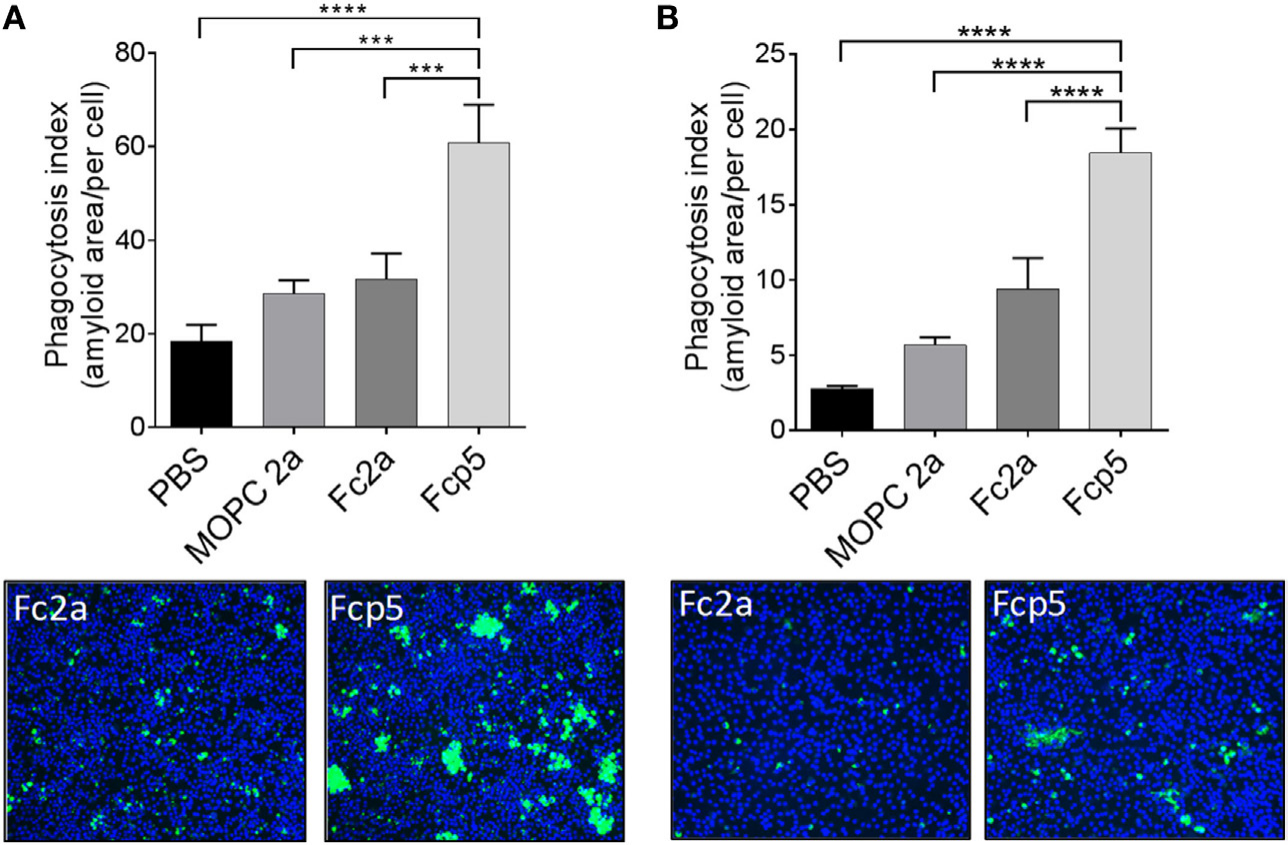
Phagocytosis of AL amyloid-like fibrils and human ATTR extracts is enhanced by opsonization by Fcp5. Quantitative estimation of phagocytosis of pHrodo green-labeled rVλ6Wil amyloid-like fibrils **(A)** and human ATTR amyloid extract **(B)**. Addition of Fcp5 significantly enhanced phagocytosis of the substrate by adherent RAW264.7 cells. Images of the engulfed (fluorescent) substrate demonstrate the qualitative difference between Fc2a and Fcp5 incubation. Original objective magnification 10×. ***p < 0.0005 and ****p < 0.0001 using ANOVA with Dunnett correction for multiple comparisons; Mean ± SD, *n* = 3.

To examine the bioactivity of the Fc component in the Fcp5 construct, ADCP was studied using an *ex vivo* phagocytosis assay. Phagocytosis of rVλ6Wil amyloid-like fibrils (Figure 5A) and human ATTR amyloid extracts (Figure 5B) labeled with the pH-sensitive dye, pHrodo green, by RAW.264.7 macrophages was measured. Labeled fibrils or ATTR amyloid extract were incubated in the presence of Fcp5 or, as negative control, MOPC IgG2a, Fc2a, or PBS alone in the presence of coverslip-bound macrophages. Cellular uptake of the labeled fibrils or extract into acidified phagolysosomes resulted in an increase in the fluorescence intensity of the pHrodo green fluorophore. The mean area of green fluorescence (*n* = 4 microscope FOV) was quantified and expressed per cell, based on a count of Hoescht-stained nuclei in each FOV (Figure 5). Using this technique, we observed a significant increase in phagocytosis of the fibrils and extracts in the presence of Fcp5, relative to all control samples. Digital images of the RAW.264.7 macrophages incubated with Fc2a- and Fcp5-treated substrate demonstrated the increased green fluorescence emission from the Fcp5 opsonized fibrils (Figure 5A) and human ATTR amyloid, relative to the control (Figure 5B).

### *In Vivo* Pharmacokinetics and Amyloid Reactivity

**Figure 6:**
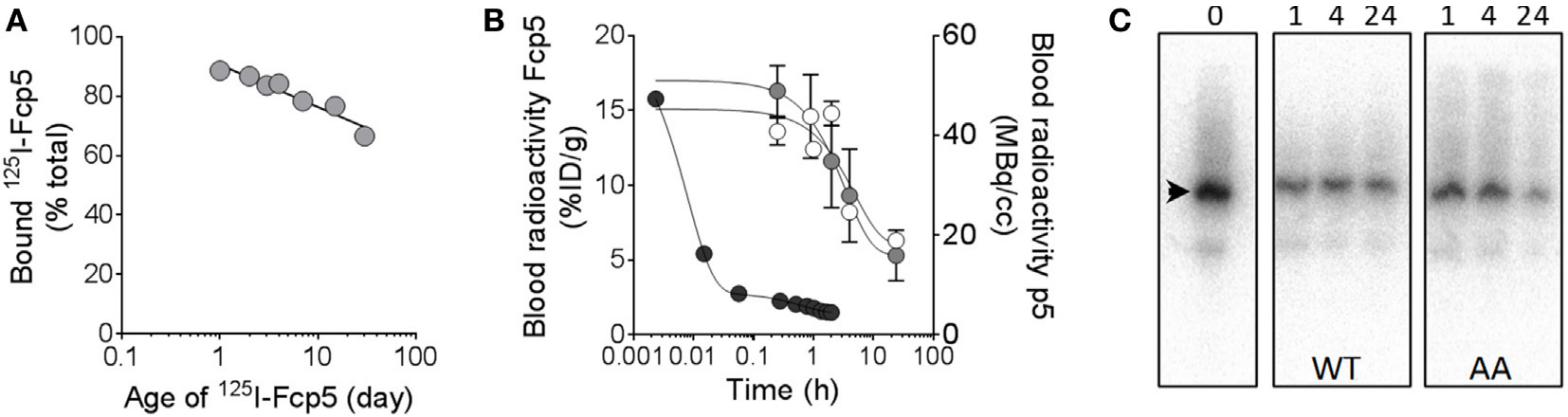
Radioiodinated Fcp5 is stable *in vitro* and *in vivo* with an extended serum half-life as compared to p5. **(A)** Binding of a preparation of ^125^I-Fcp5 to rVλ6Wil fibrils was assessed using a pulldown assay for 30 days post labeling. **(B)** ^125^I-Fcp5 in the blood of wild-type (WT) (white) and AA (gray) mice was measured (%ID/g) over 48 h postinjection by gamma counting and compared to historical data for ^124^I-p5 in WT mice (black; MBq/cc). **(C)** Serum stability of ^125^I-Fcp5 in AA and WT mice, up to 24 h postinjection, was assessed by SDS-PAGE and phosphorimaging. There was no evidence of low MW radiolabeled metabolites nor a reduction in the MW of the ^125^I-Fcp5 (arrowhead).

The *in vivo* characteristics of Fcp5 were studied following radiodionation of the protein (Figure 6). The reagent was readily radiolabeled with ^125^I with a radiochemical yield of ~50% (*n* = 3 reactions) and a radiochemical purity of >90% based on estimation of free radioiodide in the purified product by analysis of SDS-PAGE phosphor images (data not shown). The ^125^I-Fcp5, stored at 4°C in PBS with 0.1% gelatin, retained its affinity for rVλ6Wil amyloid-like fibrils, as evidenced using a pulldown assay, for more than 30 days with an estimated loss of total binding activity of only 1.8% per day over this period (Figure 6A). Estimation of the circulating half-life of ^125^I-Fcp5 was performed in healthy, WT mice, and animals with systemic AA amyloidosis by measuring the percent injected dose (%ID/g) in serum samples collected from cohorts of mice over 24 h postinjection (Figure 6B). The data were analyzed using a one-phase exponential decay equation, which yielded a serum *t*_1/2_ for ^125^I-Fcp5 in WT mice of 3.4 h (white) and in AA mice of 2.4 h (gray). The pharmacokinetics of ^124^I-p5 have been previously studied in WT mice, by using dynamic PET/CT (32), and is shown here by way of a comparison (black). Analysis of serum samples collected from WT and AA mice at 1, 4, and 24 h postinjection, by SDS-PAGE followed by phosphor imaging, indicated that the circulating ^125^I-Fcp5 was structurally intact with no evidence of radiolabeled proteolytic fragments and no indication of a truncated ^125^I-Fcp5 (Figure 6C). The gel profile for each serum sample was indistinguishable from the injected material (time 0; Figure 6C).

The biodistribution of ^125^I-Fcp5 (10 µg doped in a therapeutic IV bolus of ~4 mg/Kg of non-radiolabeled Fcp5) was studied in AA and WT mice (*n* = 3 per time point; Table 1). Consistent with the serum pharmacokinetics data, there was a high concentration of ^125^I-Fcp5 in the blood at each time point in both AA and WT animals. In the AA mice, there was evidence of retention of ^125^I-Fcp5 in the liver (~10% ID/g at 1 h postinjection), relative to the WT mice (~8% ID/g); however, this was not significantly different (Table 1).

**Table 1:** Biodistribution of ^125^I-Fcp5 in AA and wild-type (WT) mice expressed as percent injected dose per gram (%ID/g).

Tissue	1 h AA	4 h AA	24 h AA	48 h AA	1 h WT	4 h WT	24 h WT	48 h WT
Muscle	0.7 ± 0.1	0.7 ± 0.2	0.7 ± 0.1	0.4 ± 0.1	0.6 ± 0.1	0.7 ± 0.1	0.6 ± 0.1	0.6 ± 0.2
Liver	9.8 ± 2.4	6.2 ± 0.7	2.2 ± 0.3	2.4 ± 1.1	7.5 ± 0.2	4.7 ± 1.5	3.0 ± 0.3	2.6 ± 0.3
Pancreas	2.7 ± 0.4	2.2 ± 0.4	1.4 ± 0.3	1.2 ± 0.2	1.7 ± 0.4	1.1 ± 0.1	0.9 ± 0.1	0.7 ± 0.1
Spleen	4.4 ± 0.4	2.5 ± 0.5	1.4 ± 0.4	1.5 ± 0.2	6.0 ± 0.4	2.6 ± 1.3	2.3 ± 0.2	1.9 ± 0.3
Kidney	4.1 ± 0.6	3.5 ± 0.1	1.6 ± 0.3	1.5 ± 0.4	7.1 ± 0.6	4.3 ± 0.7	2.3 ± 0.4	1.6 ± 0.2
Heart	4.4 ± 0.8	3.1 ± 0.5	1.6 ± 0.7	1.3 ± 0.4	4.4 ± 0.9	3.0 ± 0.6	1.4 ± 0.1	1.4 ± 0.3
Blood	9.9 ± 3.2	6.7 ± 1.3	3.5 ± 0.1	3.1 ± 1.3	14.3 ± 1.1	8.5 ± 1.1	4.9 ± 0.3	3.8 ± 0.7

**Figure 7:**
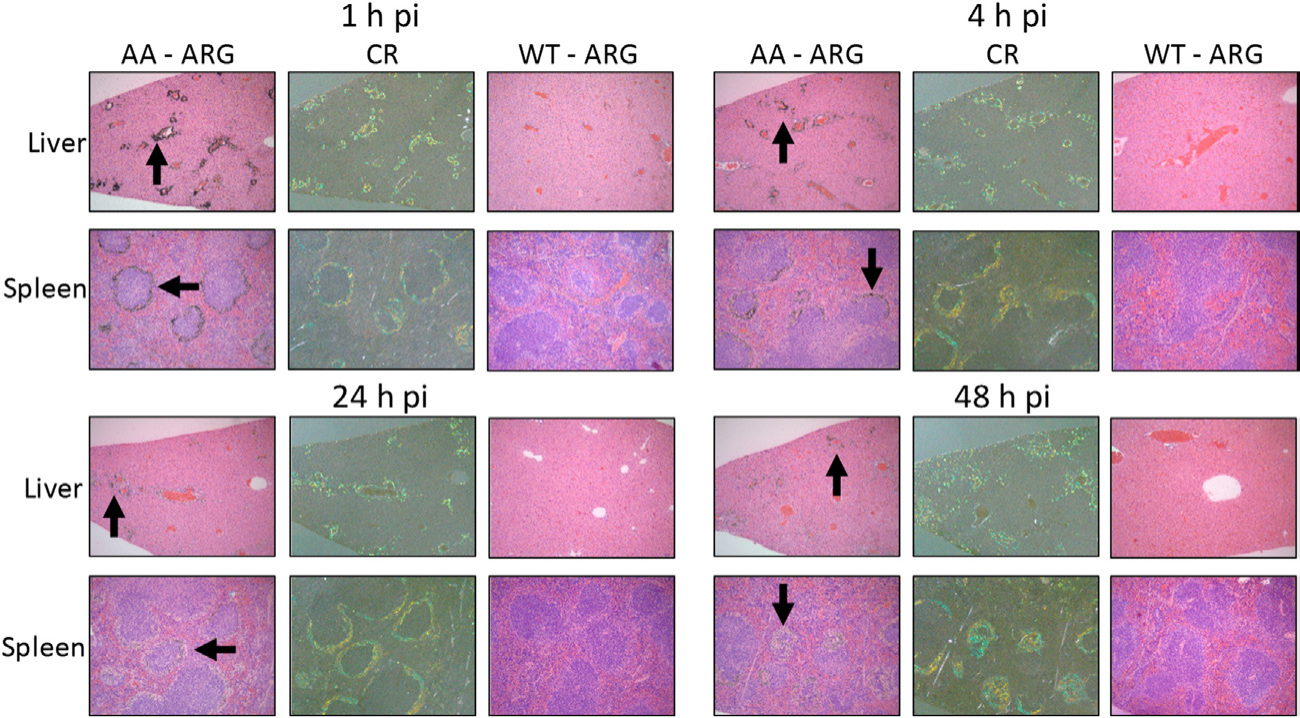
^125^I-Fcp5 binds AA amyloid deposits *in vivo*. The presence of ^125^I-Fcp5 in the liver and spleen at 1, 4, 24, and 48 h postinjection (pi) was evidenced by the presence of black silver deposits (arrows) in autoradiographs (ARG). The tissue distribution of ^125^I-Fcp5 correlated with the presence of amyloid seen as green-gold birefringent material in Congo red-stained (CR) consecutive tissue sections. No evidence of specific binding of ^125^I-Fcp5 to wild-type (WT) liver and spleen was observed.

To assess the specific reactivity of ^125^I-Fcp5 with AA amyloid deposits in the liver and spleen, the sites of major amyloid deposition in the transgenic mouse model of AA amyloidosis, tissue sections were prepared and microautoradiography performed (Figure 7). At each time point (1–48 h postinjection), there was evidence of specific accumulation of ^125^I-Fcp5 in amyloid deposits, most notably within the liver, as evidenced by the presence of black silver grains associated with the presence of radiolabeled Fcp5 (arrows, Figure 7). The deposition of ^125^I-Fcp5 correlated anatomically with sites of amyloid deposition in the organs seen as green-gold birefringent material in CR-stained consecutive tissue sections. The binding of ^125^I-Fcp5, particularly with hepatic amyloid lesions, persisted, albeit weakened, even at 48 h postinjection. The density of silver grains in the splenic amyloid deposits was less than that seen in the liver; however, specific amyloid binding of ^125^I-Fcp5 at 48 h was still detectable (Figure 7). There was no evidence, in the microautoradiographs, of specific cell-associated focal binding of ^125^I-Fcp5 in WT liver or spleen tissues (Figure 7).

**Figure 8:**
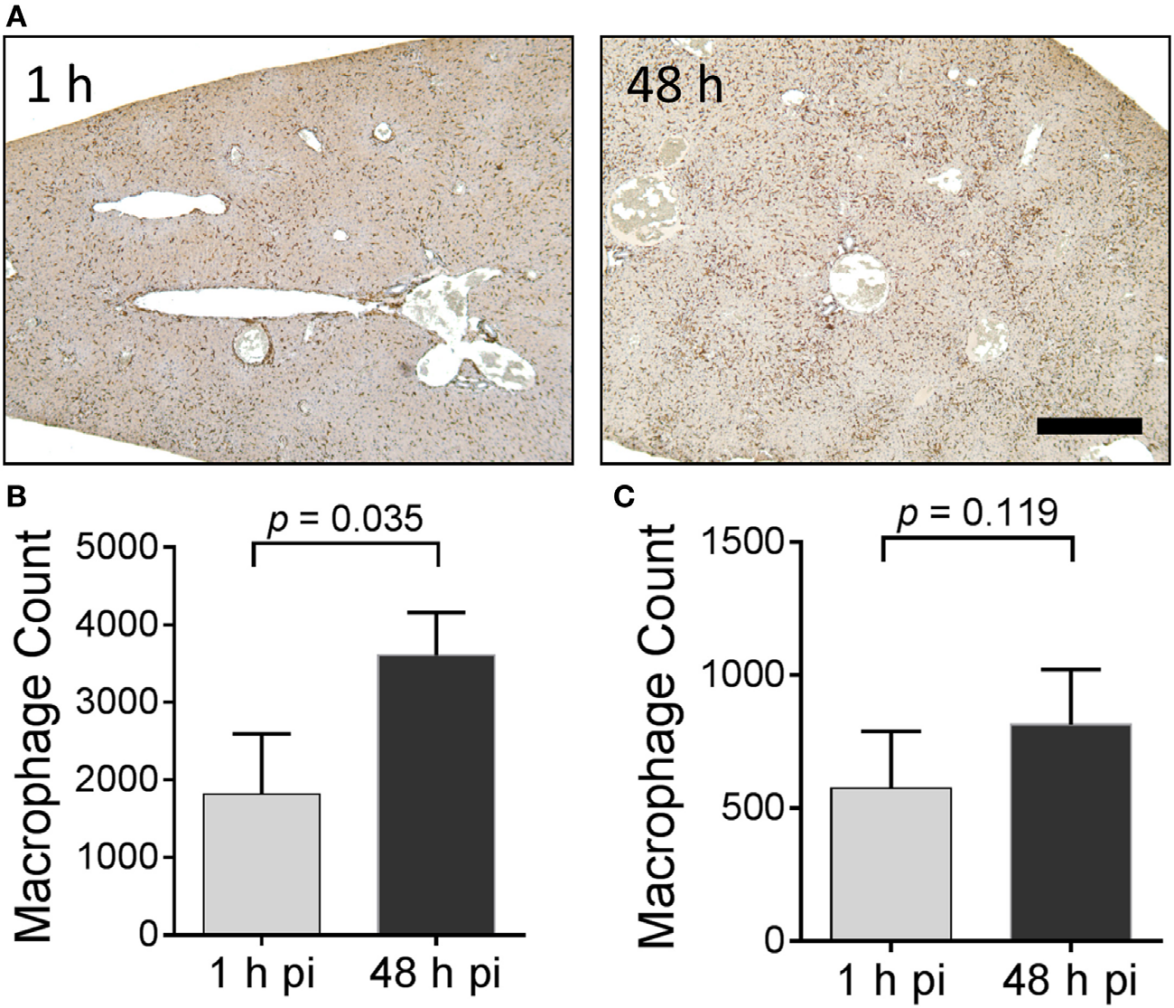
Administration of Fcp5 enhances hepatic macrophage number over 48 h in AA mice but not wild-type (WT) mice. **(A)** Formalin-fixed liver tissue from mice at 1 and 48 h postinjection of Fcp5 was immunostained with Iba-1-reactive monoclonal antibody and the number of immunopositive cells quantified by image segmentation. The bar is 300 pixels in length. Comparison (two-tailed *t*-test) of hepatic macrophage number in AA **(B)** and WT **(C)** mice at 1 and 48 h postinjection of Fcp5. Mean ± SD, *n* = 3.

The distribution of Iba-1-positive macrophages was studied microscopically in these mice (Figure 8). For this analysis, only the liver was evaluated since this organ yielded evidence of the greatest accumulation of ^125^I-Fcp5 in amyloid deposits (Figure 7). Formalin-fixed paraffin-embedded tissue sections were immunostained using an Iba-1-reactive pAb and the number of stained cells quantified by image segmentation from digital images (Figure 8A). Positively stained macrophages were more prevalent in the Fcp5-treated mice at 48 h postinjection as compared to 1 h postinjection (Figure 8A). The number of macrophages was calculated from tissue sections of three AA mice (Figure 8B) and three WT mice (Figure 8C) at 1 and 48 h postinjection. There was a significant increase in the Iba-1-positive cells in the AA liver, but not the WT liver of Fcp5-treated mice.

## Discussion

### Potential Clinical Application

Effective treatment of patients with systemic amyloidosis has, until recently, been challenging. Most therapeutic approaches have focused on reducing the concentration of the amyloid-forming precursor protein by using anti-plasma cell chemotherapy, anti-inflammatory drugs, antisense oligonucleotides, or with protein-stabilizing small molecules for AL-, AA-, and ATTR-associated amyloidoses, respectively (33–35). Patients with AL amyloidosis have benefited from stem cell transplantation and those with ATTR amyloidosis from liver organ transplantation; however, many patients present with advanced disease and are not eligible for transplantation regimens, and even following transplant, disease often recurs.

Recently, three clinical trials have begun that evaluate the therapeutic efficacy of amyloid-reactive mAbs that specifically bind and opsonize the tissue amyloid deposits. These treatments facilitate recruitment of phagocytic macrophages that mediate amyloid removal through, presumably, ADCP; moreover, evidence of mAb binding to potentially toxic oligomeric light chain species may afford an additional measure of protection (36). Interim reports from these trials have indicated improvement in patients based on positive changes in surrogate biomarkers of cardiac and renal function, N-terminal pro B-type natriuretic peptide (NT-proBNP) and proteinuria, respectively (9, 10). In one trial, which incorporates an imaging component, a reduction in hepatic amyloid load has been documented in a subset of patients by using planar gamma scintigraphic imaging of amyloid (8). Thus, passive immunotherapy with amyloid-reactive opsonizing mAbs in patients with systemic amyloidosis can yield clinical benefit.

At present, only one of the current mAbs in clinical trial has the potential to treat many forms of systemic amyloidosis due to reactivity of the mAb with the serum amyloid P component (SAP), which is ubiquitously present in all amyloid deposits (37). Similarly, Fcp5, by virtue of the ability of peptide p5 to bind universal electrostatic motifs in amyloid deposits, is capable of binding many varied amyloid deposits. However, in contrast to Fcp5, anti-SAP passive immunotherapy is a two-step process wherein circulating SAP must first be depleted by pre-treatment using a small molecule (38) while leaving the amyloid-bound SAP intact. There are no data that suggest the presence of a circulating ligand, in mice, that binds Fcp5 and competes for amyloid binding.

Furthermore, there are currently no clinical trial data indicating these mAbs will effectively opsonize leukocyte chemotactic factor 2 (ALect2)-associated amyloid, which is now the third most common form of systemic amyloidosis in the US (39). In contrast, we have shown, albeit immunohistochemically, that Fcp5 binds ALect2 amyloid deposits in formalin-fixed tissues (Figure 3).

### Pan-Amyloid Reactivity of Fcp5

To address the need for novel paradigms to treat patients with systemic amyloidosis, we have developed Fcp5, a bifunctional reagent that combines a pan-amyloid-reactive agent, peptide p5, fused with an IgG2a Fc domain. This construct was designed to enhance the circulating half-life of the peptide and generate an amyloid opsonin to mediate ADCP. This reagent has the potential to target many, if not all, forms of the systemic amyloidoses and thereby effect removal of tissue amyloid deposits (Figures 2–4). Immunoglobulin Fc-fusion proteins, sometimes termed *peptibodies*, have been developed as FDA-approved-therapeutics for numerous indications. The Fc domain, by virtue of its interaction with the FcRn and subsequent recycling, is used to extend the half-life of the associated bioactive protein, which otherwise would be rapidly catabolized. One Fc reagent, fused with the general amyloid interaction motif protein from a bacteriophage capsid protein, has been shown to bind Aβ amyloid and, when used to treat transgenic mice with cerebral Aβ amyloid deposits, showed positive therapeutic benefit (40, 41). We have previously demonstrated that peptide p5, and related polybasic synthetic peptides, bind Aβ amyloid in tissue sections as well as synthetic Aβ(1–40) amyloid-like fibrils *in vitro* (12, 13, 26). This suggests that Fcp5, delivered intravenously, may, in addition to the systemic amyloidoses, effectively target pathologic cerebral amyloid-related disorders, such as those found in patients with Alzheimer’s disease or spongiform encephalopathies (prion-related disorders).

Herein, we have demonstrated that the Fcp5 protein retains the amyloid reactivity of the parent 31-mer peptide p5 both *in vitro* and *in vivo*. Based on the estimated EC_50_ of Fcp5 for surface-adsorbed low molecular weight heparin and amyloid-like fibrils, as evidenced in EuLISA studies, the binding affinity was greater than that of the p5 peptide. This is likely due, in part, to the dimeric structure of Fcp5, which can mediate bivalent interactions and thus engage in high avidity binding to amyloid. Similar enhanced affinity has been observed for other Fc constructs where, in addition to bivalency, two or more copies of the binding motif have been incorporated into each of the Fc arms (42). In contrast to the EuLISA that favors bivalent interactions due to the high target density in the microplate well, solution-phase pulldown assays are likely governed by univalent interactions (Figures 2 and 4). When human amyloid extracts, containing, in addition to fibrils, heparan sulfate proteoglycans and other accessory molecules, were used as a substrate in pulldown assays, ^125^I-p5 bound in greater amounts as compared to ^125^I-Fcp5 (Figure 4). We hypothesize that the size of the Fc moiety may negatively impact the interaction of the Fc-associated p5 peptide in this assay. Alternatively, given that Fcp5 is 20-fold larger than peptide p5, it may reflect a decrease in the availability of binding sites due to the inability of the Fcp5 to penetrate the complex amyloid extract, as compared to the smaller peptide p5. That said, ^125^I-Fcp5 bound to all AL and ATTR amyloid extracts (with the exception of the ALκ4 amyloid, Cab) more effectively than ^125^I-11-1F4 mAb, which has been shown to image AL amyloid deposits in patients (43) and afforded clinical benefit in ~70% of enrolled subjects in the Phase 1a/b trial (11).

Effective development of peptides and polypeptides as therapeutics has been plagued by limitations due to their short circulation half-life and rapid catabolism; however, numerous strategies have been developed to overcome this problem (44). Of the potential approaches, Fc-fusion strategies have been successfully employed to address this shortcoming, and numerous reagents have been approved by the FDA for human use (45). Although our goal is to develop Fc-conjugated peptides as novel passive immunotherapeutics in a non-radiolabeled form, to study the behavior of Fcp5 *in vivo*, it was radiolabeled using ^125^I. The purified ^125^I-labeled Fcp5 retained its reactivity with amyloid-like fibrils composed of rVλ6Wil and was stable during storage for up to 30 days—fibril binding declined at only 1.8% of maximum, per day (Figure 6A). The serum half-life of ^125^I-Fcp5 was measured by following the radioiodinated reagent in the circulation over 24 h postinjection. Given that data collection was not extended to days, the estimated *T*_1/2_ of 3.4 h in WT mice likely represents only the fast (α) component of the two-phase decay curve. It is notable that even at 24 h, ~25% of the ^125^I-Fcp5 remained in the circulation at 24 h postinjection (as compared to the 5 min time point). These observations are consistent with other measurements of Fc circulating half-life where *T*_1/2fast_ (α) was ~3 h, which is followed by a *T*_1/2slow_ (β) of >100 h (46). Future studies are required that include earlier and later time points to validate this supposition. Regardless, addition of the Fc moiety extended the serum half-life of the peptide relative to that of ^124^I-p5, which was shown to have a *T*_1/2slow_ component of the bi-exponential decay of ~30 min and a peak kidney uptake time of only 7 min postinjection (32). Prolongation of the serum half-life should contribute to improved interactions of Fcp5 and similar variants with the tissue amyloid in patients.

### Opsonization and *In Vivo* Amyloid Reactivity

A critical requirement of the bifunctional Fcp5 for its use as an amyloid therapeutic, in addition to specific amyloid binding, is its ability to serve as an opsonin. We have demonstrated, using a quantitative fluorescence-based phagocytosis assay, that synthetic amyloid-like fibrils and human ATTR amyloid extracts can be effectively opsonized by pre-incubation with Fcp5, leading to enhanced uptake by surface-bound macrophages (Figure 5). Although the effector cell(s) that mediate amyloid dissolution in patients remain undefined, data suggest that macrophages and giant cells are likely involved (47, 48).

Complement fixation may have been important in the passive immuno-dissolution of amyloid *in vivo* when the SAP-reactive antibody was used (8). The fixation of complement by Fcp5 has not been evaluated and may not be an absolute requirement for ADCP of amyloidosis. Recent advances in Fc engineering have led to amino acid substituted variants and modified glycoforms that impart altered biological properties such as increased affinity for FcRn, increased complement fixation, enhanced and selective FcγR isoform binding, and decreased immunological activity [reviewed in Park et al. (18)]. Antigen-reactive Fc moieties have also been developed that incorporate paratopes engineered into the heavy constant domain 2 (CH2) to serve as truncated IgG (49). In the case of Fcp5, we have chosen to incorporate peptide p5 to function as the “paratope” and direct highly specific, high-affinity binding of the Fc to the target amyloid deposits. The initial Fcp5 protein produced in HEK cells has not been optimized for any biological function; however, this can be readily explored by using an engineered IgG1 isotype-based Fcp5 and altering the glycoform by production in CHO cells (50–52). Studies to compare the activities of HEK- and CHO-generated Fcp5 are underway.

There are currently no murine models of AL-, ATTR-, or ALect2-associated amyloidosis that effectively recapitulate the pervasive amyloid deposits seen in patients. Therefore, the murine model of AA-associated amyloidosis has become the exemplar experimental model of systemic amyloid disease used in preclinical studies. In susceptible mice, such as those expressing the pro-inflammatory human interleukin-6 transgene, AA amyloidosis can be induced by exogenous stimuli such as a suspension of preformed AA amyloid extract [AEF (31)], synthetic fibrils (53), or even pate de foie gras (54). Within weeks of induction, the mice develop severe systemic AA-associated amyloidosis predominantly in the spleen and liver, but the pancreas, kidney, adrenal, heart, and vasculature are also involved. These extensive deposits can serve as a target for the *in vivo* quantitative evaluation of amyloid-reactive reagents such as peptides (13, 14, 17) and the Fc-peptide fusion reagents. Using this experimental model, we observed specific co-localization of ^125^I-Fcp5 was observed in hepatosplenic AA amyloid deposits, as evidenced by the presence of black punctate staining in microautoradiographs that correlated with the anatomic distribution of amyloid (Figure 7). The presence of radiolabeled Fcp5 in the amyloid deposits decreased over 48 h postinjection. This contrasts with similar observations of bound half-life of radiolabeled peptides p5 and the related p5+14, which were retained at high density for more than 72 h (13). The loss of radiolabel from the amyloid-bound Fcp5 may reflect dissociation of ^125^I-Fcp5 or, alternatively, may indicate dehalogenation of the ^125^I-Fcp5 *in situ*, and subsequent loss of radioiodine to the circulation. Although we cannot definitively discern between these two scenarios, we observed a substantial influx of Iba1-positive macrophages in the livers of Fcp5-treated AA mice. Importantly, deiodinases are generally intracellular enzymes (55); therefore, if dehalogenation of amyloid-bound ^125^I-Fcp5 is occurring, it may reflect Fcp5-mediated ADCP (Figure 8). Further investigation and more direct measurement of the pharmacokinetics of Fcp5 in amyloid-laden mice and of the opsonization function *in vivo* are necessary to confirm this hypothesis.

If, however, the rapid loss of radioiodine from the amyloid deposits within 48 h postinjection reflects a short amyloid-bound half-life, improved Fc-peptide constructs may be required to enhance the peptide–amyloid interaction. To address this, we are generating reagents that increase the charge density of the amyloidophilic peptide moiety and exploring the use, either as an alternative or in conjunction with p5, of polybasic peptides with β-sheet primary structure [e.g., peptide p5_(sheet)_ (14)].

### Summary

The most important biophysical properties required of a passive immunotherapeutic for the removal of tissue amyloid are: *in vivo* stability; specific amyloid targeting; extended biological half-life; extended amyloid-bound half-life; and the ability to opsonize and recruit macrophages and induce phagocytosis of the amyloid. We have prepared a protein, Fcp5, that is stable, has 3 h serum half-life, exhibits specific, pan-amyloid reactivity, and is functional in *in vitro* phagocytosis assays. The Fcp5 specifically binds systemic AA amyloid deposits in a mouse model, and preliminary data suggest that, at least for the liver, this interaction enhances recruitment of macrophages. These data support our premise that Fc-peptide fusion proteins that incorporate synthetic amyloid-reactive peptides may serve as effective, immunologically active amyloid-targeting agents. Further optimization, by peptide or Fc engineering and expression in CHO cells, may lead to an Fcp5 variant that can serve as a beneficial, universal immunotherapeutic for patients with amyloidosis.

## Ethics Statement

All patient-derived tissue samples were used in accordance with an Institutional Review Board-approved application. Animal studies were carried out in strict accordance with a protocols approved by the University of Tennessee Institutional Animal Care and Use Committee. All procedures were approved by the IACUC and were performed in accordance with the guidelines provided by OLAW and the Guide for the Care and Use of Laboratory Animals. The University of Tennessee Medical Center animal program is AAALAC-i-accredited.

## Author Contributions

JW, AW, JF, and SK designed the experiments; JW, EM, SK, and JF analyzed the data, prepared the figures, and wrote the paper; AW, JF, AS, TR, SM, DW, RK-T, and SK performed the experiments.

## Conflict of Interest Statement

JW, JF, and SK are inventors on a US provisional patent application that describes the use of Fcp5 as a potential therapeutic opsonizing agent for amyloidosis. JW is inventor on a patent (USPTO #8,105,594) that describes the use of mAb 11-1F4 as a therapeutic agent for AL amyloidosis.
